# Prevalence and perceived importance of racial matching in the psychotherapeutic dyad: a national survey of addictions treatment clinical practices

**DOI:** 10.1186/s13011-020-00318-x

**Published:** 2020-10-08

**Authors:** Jesse A. Steinfeldt, Shondra L. Clay, Paul E. Priester

**Affiliations:** 1 Counseling and Educational Psychology Department, Indian University, Bloomington, USA; 2 School of Interdisciplinary Health Profession, Northern Illinois University, DeKalb, USA

**Keywords:** Culturally responsive counseling, Cross-racial counseling, Addictions treatment

## Abstract

**Background:**

Despite conflicting results in the literature concerning its efficacy in practice, racial matching has been identified as a component of culturally sensitive treatment.

**Methods:**

This study examined the perceived importance and prevalence of racial matching by surveying a national sample of substance use disorder (SUD) centers from the Substance Abuse and Mental Health Services Administration (SAMHSA).

**Results:**

Using univariate statistical analysis, results for the prevalence of racial matching revealed that in 58% of the clinics, there was the potential to match a counselor with a racially similar client, while in 39% of the clinics, there was no potential to provide such a match. Among the agencies that displayed a potential for racial matching, 26% of the respondents indicated that they never racially matched clients and therapists, 71% reported that they sometimes practice racial matching, 15% indicated that they usually racially match, and only 7% purported to always racially match clients and therapists. Results for the perceived importance of racial matching revealed that in both situations where treatment centers had the potential for racial matching and did not have the potential for racial matching, supervisors reported that it was relatively important to provide culturally sensitive treatment but that it was not as important to match clients in SUD centers with racially/ethnically similar counselors.

**Conclusion:**

The topic of racial matching can be very complex and has shown variation amongst SUD centers; however, this study emphasizes the importance of providing culturally sensitive treatment and an appreciation of differences among members within each racial group.

## Background

The provision of effective psychotherapeutic services is a constantly evolving process. This process is challenged by the demands of meeting the needs of a clientele that is growing increasingly ethnically diverse [1]. Acknowledging the increased demands, it is important to consider cultural considerations in psychotherapeutic services [2]. It has been noted that clients of color have substantial barriers to seeking psychological services [3–5], are less likely to receive expected benefits of counseling, report poorer quality of care [6, 7], and are at a greater risk of prematurely discontinuing counseling services [8–10]. Furthermore, low-income ethnic minority individuals have accessibility barriers that decrease the likelihood of seeking and completing mental health treatment [11–13].

Although low socioeconomic status has been often referred to as one of the major contributors to societal inequalities between different ethnic groups, disparities in health care have been documented even when controlling for socioeconomic status [14–16]. Particularly, many recent studies have posited disparities in treatment for substance use disorders (SUD) [17] including unmet needs [18] and disparities in matriculating through programs [2]

In the mental health field, one resolution that has been proffered in response to this quandary involves matching client and therapist in terms of shared race/ethnicity, which has been broadly defined as “racial matching” [19]. There are primary, “first order” treatment predictors such as therapeutic alliance [20] and the client’s relative status in the stages of change process [21]. Racial matching can be perceived as a secondary, but important component to the treatment process. Racial matching has been identified as one component of culturally sensitive treatment [22–25]. More specifically, research has supported matching services to need in SUD treatment, particularly for racial/ethnic groups [26]. However, throughout the human-service literature, considerable debate exists concerning the potential benefits and limitations associated with matching clients and psychotherapists in terms of shared ethnicity [23, 27, 28].

The belief in the efficacy of racial matching has roots in social psychological literature, which indicates that people tend to identify with individuals similar to themselves [29]. This literature base further suggests that ethnic preferences may be based on the perception that similar appearances indicate similar attitudes [30]. Psychotherapy research has shown that clients prefer a counselor whose ethnicity matches that of their own, especially ethnic minority clients [31–33]. Specifically, African American clients may prefer to see African American therapists as opposed to seeing Caucasian therapists [30, 33–35]. Furthermore, ethnic differences between client and therapist contribute to attrition, especially for Caucasian therapists who are working with ethnic minority clients [33, 36].

Despite these findings, empirical support for the practice of racial matching has yielded at best, inconclusive results, and at worst, contradictory results. In individual studies, racial matching has been demonstrated to be a viable means of enhancing the psychotherapeutic experience in terms of clients experiencing more favorable clinical outcomes [37], receiving more favorable Global Assessment of Functioning Scores [38], and attending more clinical sessions [39]. While these findings support the practice of racial matching as a means of improving client experience, other research findings do not maintain this assertion [36, 40].

Historically, there have been a number of analyses that synthesized the findings of studies conducted on racial matching. Both Maramba and Hall [24] and Shin et al. [41] have conducted meta-analytic reviews on racial matching in psychotherapy, while Karlsson [42] provided a qualitative overview of racial matching findings with the inclusion of a discussion of methodological and conceptual issues. More recently, Cabral & Smith [43] and Smith & Trimble [33] have explored racial/ethnic matching in mental health services through a meta-analytic review. These studies have attempted to consolidate the literature base and shine a more definitive light on the empirical support (or lack thereof) for racial matching.

Similar to the meta-analytic reviews, there have been several contending research findings in the literature. In a study exploring the impact of racial matching in SUD centers, Marsh et al. [26] posits that need-service matching is more effective than racial matching in SUD centers. Other studies have found that racial matching has an impact on treatment outcomes of the client [36, 44].

These reviews further illustrate the inconclusive and often contradictory empirical support for the practice of racially matching clients and therapists in psychotherapy. The reviews support that racial matching may be a viable practice for enhancing the psychotherapeutic experience for clients; however, there are important considerations. The methodological and conceptual issues are hampering the emergence of empirical support.

The topic of racial matching has been unequivocally complex. Examining SUD clinics at the national level using the Substance Abuse and Mental Health Services Administration (SAMHSA), this study examined the perceived importance and prevalence of racial matching. More specifically, this study aimed to ascertain the perception of importance placed on providing culturally sensitive treatment, based on the contention that racial matching represents a component of culturally sensitive treatment [22, 24, 33]. The guiding research questions are intended to: (1) discern the importance that these clinics placed on racial matching of clients and therapists, and (2) explore the prevalence rates of racial matching practices, as well as the degree to which these centers have the potential to engage in this practice, if in fact they are.

## Methodology

### Participants

Two hundred and forty SUD treatment centers were randomly selected from the Substance Abuse and Mental Health Services Administration (SAMHSA) Treatment Provider Directory. A stratified sampling technique was employed in order to ensure representation from all regions of the country. SAMHSA lists SUD treatment centers by geographical regions. We selected 25% (*n* = 60) treatment centers from each of the four regions (East, South, Midwest and West). The treatments centers were randomly selected from a hard copy of the SAMSHA guide to treatment provider. The authors contacted each treatment center via telephone to identify the name of the clinical supervisor. All of the information regarding the demographics of the treatment centers and related clinical practices were obtained from this clinical supervisor. A clinical supervisor was considered the best local source of accurate information, given their supervisory role and larger systems perspective of the specific treatment center.

### Response rate

One hundred and thirty nine of the 240 surveys were returned for a response rate of 58%. Thirty-nine of the surveys were returned with incomplete information and were excluded from analyses. The authors consider this to be an acceptable rate, given the overstressed nature of the population sampled.

To increase the response rate and according to typical survey response procedures, the participants were informed that, if they so desired, they could include a business card with the individual’s favorite charity written on back. One business card would be randomly drawn from these and a $100 donation would be donated to that charity. A graduate student not associated with the rest of the analysis had the responsibility of opening the received envelopes and retrieving the business card (if one were present). In this way, there was no way of identifying any of the responses with specific individuals. The randomly drawn business card asked that a donation be made to the local Humane Society. This donation was then made.

### Instrument

The authors developed a survey for the purposes of this study. This survey asked, with a four-point Likert-type question, whether the clinical supervisors believed that it is important to match clients with racially/ethnically similar counselors. An additional four-point Likert-type item inquired whether the clinical supervisor felt that it was important to provide culturally sensitive treatment. Other items on the survey inquired about demographic information on the counselors and clinical population served at the agency, thoroughly examining the relative proportions of racial/ethnic composition of clients and clinicians.

### Procedure

Once the completed surveys were analyzed, the authors carefully selected treatment centers where it was possible to provide a racial match. Accordingly, if there was just one counselor who shared a self-reported racial status with at least one client, the potential was seen as having been existent, and the survey was included in the analysis. Note, there could be the situation in which there was an African American clinician and no African American clients, but there were Latinx clients. In this situation, it was determined that this clinical setting did not provide the opportunity for a racial match because this study intended to examine specifically matching similar race/ethnicities in the psychotherapeutic dyad, not the potential to match non-majority clinicians with non-majority clients.

### Analysis

Of the treatment centers where there was the possibility of a racial match, a chi-square analysis was completed comparing the responses on the two Likert-type items: “How important is it to match clients with racially/ethnically similar counselors?” and “How important is it to provide culturally sensitive treatment?” Also, multinomial logit modeling was used to determine the likelihood of the potential to racially match. Analyses were performed using IBM SPSS v. 25.0.

## Results

### Demographic information

#### Treatment centers and clinical staff

There was a healthy degree of heterogeneity in the demographics associated with the sample treatment centers and the clinicians working at these facilities. A complete description of the demographics of the SUD treatment centers and the clinicians that are employed is listed in Table 1.

**Table 1:** Demographics of Sampled Treatment Centers

Treatment modality	
Exclusively inpatient	13% (*n* = 18)
Exclusively outpatient	55% (*n* = 76)
Both inpatient and outpatient	32% (*n* = 44)
Client population served	
Exclusively adults	55% (*n* = 76)
Exclusively adolescents	4% (*n* = 6)
Both adolescents and adults	45% (*n* = 63)
Size of client population	
Number of clients served in a typical month	Between 4 and 2000; $\overline{\boldsymbol{x}}$ =194.36, SD = 301.89
Organizational affiliation	
Affiliated with a hospital	13% (*n* = 19)
Affiliated with a correctional system	3% (*n* = 4)
Affiliated with a private mental health practice	22% (*n* = 30)
Affiliated with a faith-based organization	3% (*n* = 4)
Affiliated with the government	11% (*n* = 15)
Affiliated with a community-based organization	39% (*n* = 54)
No stated affiliation	4% (*n* = 5)
Counselor demographics	
Number of full-time counselors employed	Between 1 and 120; $\overline{\boldsymbol{x}}$ =9.89, SD = 14.56
Number of part-time counselors	Between 0 and 15; $\overline{\boldsymbol{x}}$ =3.06, SD = 3.02
Percent of counselors in recovery from addictive disorders	Between 0 and 100%; $\overline{\boldsymbol{x}}$ =48%, SD = 30
Minimal educational requirements for clinicians	
No minimum educational requirements	10% (*n* = 14)
High school diploma	24% (*n* = 33)
Associate’s degree	6% (*n* = 8)
Bachelor’s degree	43% (*n* = 60)
Master’s degree	7% (*n* = 8)
Doctorate degree	2% (*n* = 3)
Did not answer question	7% (*n* = 10)
Certification requirements for clinicians	
Provisional SAC Certification	32% (*n* = 44)
SAC certification	44% (*n* = 61)
No certification required	16% (*n* = 23)
No response	8% (*n* = 11)

#### Racial/ethnic composition

The race of the counselors was: 74% European American, 20% African American, 1% Native American, < 1% Asian American and < 1% “other.” The race of clients receiving services at the surveyed clinics was: 65% European American, 22% African American, 8% Latino, 3% Native American, 2% Asian American and < 1% “other.”

#### Potential for racial match

In 58% of the clinics, there was the potential to match a counselor with a racially similar client, while in 39% of the clinics, there was no potential to provide such a match. To determine whether there was a potential for racial match, the racial demographics of the clients were compared to the racial demographics of the counselors. For example, if the client demographics of a specific treatment center included 30% African American clients and the counselor racial demographics of that site included 10% of the clinicians being African American, this was identified as a site that had the potential for racial matching. If a particular site had a client racial demographic profile of 50% Latino and 50% White and the counselor racial profile was 80% White and 20% African American, this would be considered as a site where no racial matching was possible. Of the agencies sampled, 47% of clinics had the potential to match African American clients with African American counselors, 28% of those agencies had the potential to match Latino/a clients with racially similar counselors, 8% had the potential to racially match Native American clients with Native American counselors, and only 2% of clinics had the potential to racially match Asian American clients with Asian American counselors.

#### Prevalence of racial match

Among the agencies that displayed a potential for racial matching, 26% of the respondents indicated that they never racially matched clients and therapists, 71% reported that they sometimes practice racial matching, 15% indicated that they usually racially match, and only 7% purported to always racially match clients and therapists.

#### Responses to “How important do you think it is to provide culturally sensitive treatment?”

For the agencies at which there was a potential for racial matching, 72% of respondents indicated that they felt that it was very important to provide culturally sensitive care, while 22% indicated that it was somewhat important, 2% reported that it was only slightly important, and 3% reported that it was not important. Two percent of respondents did not provide a response to this item. For agencies at which there was no potential racial matching, 61% of respondents indicated that they felt that it was very important to provide culturally sensitive care, while 24% reported that it was somewhat important, 13% reported that it was slightly important, and 3% reported it was not important.

#### Responses to “Do you think that it is clinically important to match clients withracially/ethnically similar counselors?”

When there was a potential for match, 16% of respondents indicated that they thought it was important to match clients with racially/ethnically similar counselors, while 26% reported that it was somewhat important, 36% reported that it was only slightly important, and 22% reported that it was not important. When there was no potential for racial matching, 5% of respondents indicated that they thought matching clients with racially/ethnically similar clients was very important, 45% reported that it was somewhat important, 37% reported it was only slightly important, and 13% reported it was not important.

##### Analyses

In the situation where treatment centers had the potential for racial matching, the difference between the two Likert-type items was significant, *χ*^2^(3, *n* = 58) = 47.74, *p* < .001. Supervisors reported that it was relatively important to provide culturally sensitive treatment but that it was not as important to match clients with racially/ethnically similar counselors.

In the situation where treatment centers did not have the potential for racial matching, the difference between the two Likert-type items was significant, *χ*^2^(3, *n* = 38) = 27.03, *p* < .001. Once again, supervisors reported that it was relatively important to provide culturally sensitive treatment but that it was not as important to match clients with racially similar counselors.

Binary logistic regression models were performed to analyze predictors of potential to racially match based on other characteristics associated with racial matching. The first model was employed on all variables and yielded a Cox & Snell *R*^*2*^ = .704. No results were statistically significant. However, the second model (Cox & Snell *R*^*2*^ = .244) removed variables that were not empirically supported by the association of racially matching (e.g. minimum counselor education, percent of counselors and clients who are minority) to assess predictors. Results yielded a statistically significant association (OR = .411, *p* = .007) for client population (i.e. adult or adolescent population of clients). There was a statistically significant difference in ability to racially match clients with similar counselors when the client population served by the treatment center was adolescents as opposed to those serving adult clients. There was less ability to racial match psychotherapeutic dyads in the adolescent serving facilities.

## Discussion

These data indicate that the majority of administrators surveyed at the respective SUD centers in this study believe that it is very important to provide culturally sensitive treatment. However, results revealed that it was not as important to match clients with racially/ethnically similar counselors in SUD centers. Despite the contention that racial matching is commonly practiced in psychotherapy and case management services [33, 45], the SUD centers surveyed do not appear to prioritize the implementation of this practice.

On the surface, these results appear to be paradoxical. If racial matching is considered to be one component of culturally sensitive treatment, why do clinicians who strive to provide culturally sensitive services not value racial matching? One explanation may be that, despite some research support, racial matching is not fully recognized as a stand-alone mechanism of change in SUD centers particularly. Even though in generalized therapy, clients may prefer racially matching a therapist similar to their own race [33], in SUD treatment centers, racial matching may contribute to positive research outcomes as a mediating variable, as an adjunct, or in another fashion. It is possible that, without this componential acknowledgement, racial matching may not be given due regard based on a lack of clear empirical support in the literature. Thus, as pointed out by Karlsson [42], the potential benefits of racial matching may be undermined by conceptual and methodological issues that cause it to be perceived by clinicians in the field as relatively ineffective or even unimportant. Further research should address this issue.

The confounding nature of racial matching in SUD centers may be further evidenced in the distinction between racial matching and cultural matching. Sue and Zane [46] purported that, when compared to racial match, cultural match is a better predictor of treatment outcome because it is more proximal to therapy. Opposed to general therapy where some clients may value the racially matched experience to build a stronger therapeutic relationship experience [47, 48], clients who are in SUD centers may find more value in compatibility/experiences than simply possessing shared racial traits. This assertion is supported by research suggesting that racial matching differs based on context. In some instances, racial minority clients may seek other counselor characteristics over racial similarity, such as similar values, attitudes, or personalities [49]. Similarly, Horst et al. [50] acknowledged that the impact of racial matching may differ based on contextual elements. Even though racial matching may not be deemed a necessity in SUD center, it is important to acknowledge the consideration of the context.

Furthermore, rather than examining racial match exclusively, research looking to enhance client psychotherapy experiences in SUD centers may be better served by investigating certain within group variables such as cultural attitudes, racial identity, social class, cultural commitment, language, and acculturation [42]. This perspective is aligned with theoretical and empirical research that undergird group identity development frameworks [51]. Group identify status developmental framework implores that individuals, whether it is in-group or out-group, are unified by commonalities such as racial identity, shared cultural beliefs, attitudes, and/or interests. In SUD centers particularly, individuals in therapy may be less interested in racial matching and more interested in shared experiences, aligned with theoretical orientations from group identity development frameworks.

Racial identity matching differs from racial matching in that racial identity includes more than visible physical traits. By providing a more comprehensive assessment of the significance, meaning, and function of race/ethnicity, racial identity is considered to be a more relevant multidimensional psychological construct that can account for a greater understanding of social dyadic processes than the less complex demographic variable of race/ethnicity [52]. Racial identity acknowledges individuals’ psychological processes within a sociopolitical and cultural environment wherein power is differentiated by race [53–55].

As relating to the quality of one’s identification with his or her racial group and as a sense of collective identity based on a perception of common racial heritage, racial identity is used to describe and measure within-group variability of individual ethnic groups [56]. Based on this assertion, simply racially matching a client with a therapist in SUD centers may not provide instant compatibility because of the wide within-group variability. This may potentially account for the differences found in the use of racial matching among the administrators at the SUD centers. However, the findings of this study in SUD centers confirms the complexity of racial matching as contextually, there may be benefits to client/therapist didactic relationships based on race [33].

### Limitations

While this study intended to examine the state of racial matching in the SUD field, it did not break down racial matching in terms of its within group variables, particularly the aspect of acculturative language discrepancy. Shin et al. [41] purported that racial matching may matter most when there is a linguistic component. This is particularly important for SUD centers as the functional nature of language may supersede issues of preference and compatibility in terms of enhancing the psychotherapeutic experience for clients. This important aspect of racial matching warrants individual attention.

A second limitation of this study is the response rate of those SUD clinics surveyed. Although a 58% return rate is appropriate given the high stress and workload of the clinical supervisors in these settings, the supervisors who did take the time to complete the mail survey may not be representative of the entirety of the field. Similarly, the issues of power and measurement validity were not adequately addressed. Thus, the results should be interpreted within this given context and further research should utilize multiple methods to attempt to incorporate an entirely representative sample as well as reduce psychometric limitations within the study.

A final limitation is that there could be within group differences unrelated to race that could play a role in clinician assignment to specific clients. A pertinent example for this sample could be the recovery status of the clinician or previous drug of choice for the recovering clinician. Although research suggests that a client’s preference for a similarly recovering clinician is less salient during treatment and becomes more salient as the disability identity of the individual grows over time, there could be a perception that it may be more clinically powerful to match a counselor who is a recovering intravenous cocaine addict with a similar client than to match the dyad on ethnicity or stage of racial identity development [57].

## Conclusion

### Implications

This study reviewed the inconclusive nature of the racial matching literature, examined the perceptions and prevalence of racial matching in SUD treatment centers, and offered suggestions as to how racial matching can be a confounded and misunderstood construct. Culturally sensitive treatment needs to be aware of differences between racial groups, but it also requires an appreciation of differences among members within each racial group. Clinicians may not want to totally abandon matching client and therapist of similar race; rather, they should view racial matching in a multidimensional manner wherein it has the potential to be an aspect of providing culturally sensitive treatment, but other components need to be considered.

The lack of clear empirical support for racial matching highlights the importance of multicultural training for therapists of all ethnicities so therapists can deliver culturally competent psychotherapeutic services [24, 33]. Although racial matching may have an initial appeal to clients, it is the quality of respect and connection felt by the client that supersedes racial differences or similarities [36]. Through proper multicultural training, therapists aspire to transcend demographic variables and make deep and meaningful connection with all clients.

## Data Availability

Copies of the survey instrument used in this study are available by request.

## Abbreviations

SAMHSA: Substance Abuse and Mental Health Services Administration
SAC: Substance Abuse Counselor
SUD: Substance Use Disorder
